# Histone Deacetylase Inhibitors (HDACi) Promote KLF5 Ubiquitination and Degradation in Basal-like Breast Cancer

**DOI:** 10.7150/ijbs.65322

**Published:** 2022-02-28

**Authors:** Yanjie Kong, Wenlong Ren, Huan Fang, Naseer Ali Shah, Yujie Shi, Dingyun You, Chengang Zhou, Dewei Jiang, Chuanyu Yang, Huichun Liang, Wenjin Liu, Luzhen Wang, Wenqiang Gan, Xing Wan, Fubing Li, Zhen Li, Ceshi Chen, Ni Xie

**Affiliations:** 1 Biobank, The First Affiliated Hospital of Shenzhen University, Shenzhen Second People's Hospital, Shenzhen, 518035, China; 2 Key Laboratory of Animal Models and Human Disease Mechanisms of Chinese Academy of Sciences and Yunnan Province, Kunming Institute of Zoology, Kunming, Yunnan, China; 3 Kunming College of Life Sciences, University of Chinese Academy Sciences, Kunming, Yunnan, China; 4 Department of Pathology, Henan Provincial People's Hospital, Zhengzhou University, Zhengzhou, Henan, 450003, China; 5 Kunming Medical University, Kunming, Yunnan, 650500, China; 6 Department of Dermatology, Jingmen No.1 people's Hospital, Jingmen, Hubei, 448000, China; 7 Department of the Third Breast Surgery, the Third Affiliated Hospital of Kunming Medical University, Kunming, Yunnan, 650118, China; 8 School of Life Science, University of Science & Technology of China. Hefei, Anhui, 230026, China; 9 Affiliated Cancer Hospital & Institute of Guangzhou Medical University, Guangzhou, 510095, China

**Keywords:** Histone deacetylase, HDAC1, trichostatin A, suberoylanilide hydroxamic acid, basal-like breast cancer, KLF5

## Abstract

Basal-like breast cancer (BLBC) accounts for approximately 15% of all breast cancer cases, and patients with BLBC have a low survival rate. Our previous study demonstrated that the KLF5 transcription factor promotes BLBC cell proliferation and tumor growth. In this study, we demonstrated that the histone deacetylase inhibitors (HDACi), suberoylanilide hydroxamic acid (SAHA), and trichostatin A (TSA), increased KLF5 acetylation at lysine 369 (K369), downregulated KLF5 protein expression levels, and decreased cell viability in BLBC cell lines. HDACi target KLF5 for proteasomal degradation by promoting KLF5 protein ubiquitination. K369 acetylation of KLF5 decreases the binding between KLF5 and its deubiquitinase, BAP1. These findings revealed a novel mechanism by which HDACi suppress BLBC, and a novel crosstalk between KLF5 protein acetylation and ubiquitination.

## Introduction

Globally, breast cancer is consistently the most common cancer type in women, as well as the leading cause of cancer death in this group 1. Based on gene expression levels, breast cancer can be classified as luminal A, luminal B, HER2 positive, and basal-like subtypes 2. Among these, basal-like breast cancers (BLBCs) are mostly ERα, PR, and HER2 triple-negative breast cancers (TNBCs), which comprise 15-20% of all breast cancer diagnoses and are associated with poor prognosis, high risk of distant recurrence, and low five-year survival 3. Therefore, there is an urgent need to develop effective targeted therapies for BLBC.

Histone deacetylase (HDAC) inhibitors (HDACi) have emerged as a promising new class of multifunctional anticancer agents with the ability to suppress cancer cell migration, invasion, metastasis, and angiogenesis 4. More than 15 HDACi have been tested in preclinical and early clinical studies 5. Among these, vorinostat and panobinostat (LBH589; NVP-LBH589) have been approved by the FDA to treat cutaneous T-cell lymphoma and multiple myeloma, respectively 6. We previously reported that trichostatin A (TSA) and suberoylanilide hydroxamic acid (SAHA) induce growth arrest and apoptosis in TNBC 7. TSA selectively and consistently inhibited the proliferation of TNBC cell lines HCC1806 and HCC38 by inducing cell cycle arrest and apoptosis 8. SAHA in combination with epigallocatechin-3-gallate suppressed breast cancer cell growth 9. HDACi (SAHA or belinostat) reduced homologous recombination efficiency in TNBC, and sensitized TNBC to poly-ADP ribose polymerase (PARP) inhibition 10. Panobinostat and salinomycin efficiently inhibited HCC1937 cell stemness, proliferation, survival, and migration 11. Panobinostat inhibited epithelial-mesenchymal transition (EMT) and metastasis of MDA-MB-231 12. However, the mechanism by which TSA and SAHA suppress TNBC remains unclear.

The Krüppel-like factor 5 (KLF5) transcription factor is highly expressed in BLBC and induces a number of downstream target genes, such as *CCND1* 13,* FGF-BP1* 14, *mPGES1* 15, *TNFAIP2* 16, *Slug* 17, and *IGFL1-AS1* and *IGFL1* 18 which are associated with cell cycle, survival, migration, and stemness. Previous studies have shown that high expression levels of KLF5 are significantly associated with an increased risk of recurrence and poor prognosis in breast cancer patients 19. KLF5 is a potential therapeutic target for BLBC 20. KLF5 depletion suppresses BLBC initiation, growth, and metastasis 17, 21. Furthermore, inhibition of KLF5 by mifepristone and its derivatives 22-24, metformin 25, mithramycin A 26, super-enhancer inhibitors 27, and the protein arginine N-methyltransferase 5 (PRMT5) inhibitor, PJ-68 28, suppress BLBC stemness and tumor growth.

KLF5 can be acetylated 29. In response to transforming growth factor β, the lysine-acetyltransferase, p300, acetylates KLF5 at K369 to facilitate *p15* gene transcription and inhibit *Myc* gene transcription 30, 31. KLF5 acetylation at K369 plays an important role in regulating prostate development and regeneration 32. KLF5 can be deacetylated by HDAC1/2 and SET, which inhibits its function 33, 34. HDAC4 promotes airway inflammation and remodeling by deacetylating KLF5, increasing KLF5 transcriptional activity, and facilitating the progression of asthma 35.

KLF5 is an unstable protein that undergoes ubiquitin-mediated proteasomal degradation 36. E3 ligases WWP1 37, Smurf2 38, and SCF^Fbw7^ 39 have been shown to promote KLF5 ubiquitination and degradation. YAP and TAZ protect KLF5 from WWP1-mediated degradation 40, 41. PRMT5 also stabilizes KLF5 by preventing KLF5 from SCF^Fbw7^-mediated degradation 28. Three deubiquitinases (DUBs), including BAP1 21, ATXN3L 42, and USP3 43 stabilize the KLF5 protein in BLBC.

Since both acetylation and ubiquitination occur at lysine residues, it is inevitable that there is crosstalk between two posttranslational modifications. It has been reported that acetylation could either block 44, 45 or promote 46, 47 ubiquitination. A recent study suggested that KLF5 acetylation may regulate KLF5 protein stability and that knockdown of HDAC1/2 in DU145 prostate cancer cells upregulated KLF5 protein acetylation and expression levels by blocking its degradation 34. However, the mechanism by which KLF5 acetylation regulates its degradation is unclear.

In this study, we demonstrated that TSA and SAHA significantly downregulated the expression of KLF5 protein and suppressed the viability of BLBC cell lines. HDACi promoted the acetylation of KLF5 at K369, which decreased the association between BAP1 (a DUB) and KLF5. Thus, KLF5 acetylation promotes ubiquitination and proteasomal degradation in BLBC. Our findings shed light on the crosstalk between acetylation and ubiquitination of KLF5 and suggest that HDACi could be used to treat KLF5-positive BLBC.

## Materials and Methods

### Cell lines, antibodies, and reagents

MCF10A, HCC1937, HEK293T, and HCC1806 cell lines were purchased from the American Type Culture Collection (ATCC, Manassas, VA, USA). TSA, SAHA, MG132, and cycloheximide (CHX) were purchased from Sigma (St. Louis, MO, USA). Total acetylated KLF5 was detected by western blotting using an anti-acetylated lysine antibody (Minneapolis, MN. Cat# AF3758); Ac-KLF5K369 (1:1000 in 5% BSA) antibodies were generated in previous studies 36, 48. The anti-human FGF-BP1 (MAB1593) antibody was purchased from R&D Systems (Minneapolis, MN, USA). Antibodies against HDAC1, acetylated lysine, Cyclin D1, p21, Flag, Ub, c-Myc, PARP, Caspase-3, Caspase-7, β-actin, and glyceraldehyde 3-phosphate dehydrogenase (GAPDH) were purchased from Cell Signaling Technology (Danvers, MA, USA). The siRNA target sequences for the human *HDAC1* gene were HDAC1si#1 (5′-GCGACTGTTTGAGAACCTT-3′), HDAC1 siRNA #2 (5′-GGGATCGGTTAGGTTGCTT-3′), and HDAC1 si#3 (5′-AGGCGGTGGTTACACCATT-3′).

### Stable KLF5, KLF5^K369R^, and KLF5^K369Q^ expression

Full-length wild-type human *KLF5* was cloned into the pcDNA3.1 and pCDH vectors. The acetylation-deficient mutant KLF5^K369R^ (KR) and the acetylation-mimicking mutant KLF5^K369Q^ (KQ) were generated by PCR-based mutagenesis, and confirmed by sequencing. Lentiviruses expressing *KLF5* and its mutants were packaged and applied to infect MCF10A, HCC1937, and HCC1806 cells.

### Western blotting

Western blotting was performed using a previously established procedure, and the KLF5 antibody was generated as described in our previous study 36. After treatment, the cells were washed three times with 1×PBS and lysed with lysis buffer for 30 min. The cell lysate was centrifuged at 12,000 rpm for 10 min to collect the supernatant. Protein concentrations were detected using the Bradford assay (Bio-Rad Laboratories, Inc., Hercules, CA, USA). Equal amounts of proteins for each treatment were loaded and separated by SDS-PAGE, transferred to polyvinylidene fluoride membranes (GE Healthcare, Chicago, IL, USA), blocked with 5% skim milk in 1×TBST for 1 h at room temperature, and subsequently incubated overnight at 4 °C with primary antibody. After washing three times with 1×TBST, the membrane was exposed to secondary peroxidase-conjugated antibody and visualized using western blotting detection reagents (GE Healthcare). GAPDH or β-actin was used as loading control.

### Cell viability measurement

Cell proliferation in HCC1937 and HCC1806 cell lines was measured using a sulforhodamine B (SRB) assay (Sigma). Briefly, cells were seeded into 96-well plates at 5,000 cells/well. After 24 h, the cells were treated with TSA or SAHA at the indicated concentrations, and equivalent DMSO served as a negative control. The cells were then fixed with 100 μL of 10% trichloroacetic acid for 60 min; subsequently they were washed five times with deionized water. The cells were stained with 100 μL 0.4% (w/v) SRB in 1% acetic acid for 5 min, and then the plates were washed five times with 1% acetic acid, and dried. Finally, 100 μL of 10 mM Tris base was added to each well. Optical density at 540 nm was measured using a spectrophotometric plate reader. The cell viability values at different drug dosages were plotted in 2010 Excel (Microsoft, WA, USA); IC_50_ values were obtained from the graphs.

### Ubiquitination assays

Stable overexpression KLF5-3×Flag HCC1937 or HCC1806 cells were treated with TSA (1 μM) or SAHA (5 μM). After 2 days, the cells were treated with 20 μM MG132 for 6 h to enrich polyubiquitinated KLF5 proteins. The cells were harvested in lysis buffer (50 mM Tris-Cl and 1.5% SDS; pH 6.8) using a six-well plate. Each well contained 150 μL of lysis buffer. The cell lysate was boiled for 15 min to denature the proteins. BSA buffer (50 mM Tris-Cl, 180 mM NaCl, 0.5% NP-40, and 0.5% BSA; pH 6.8; 1.2 mL) was added to dilute the samples. Flag-M2 beads (30 μL per sample; prewashed with BSA buffer three times) were added to immunoprecipitate Flag-KLF5 overnight with rotation in a cold room (4 °C). The beads were washed three times with 1 mL ice-cold BSA buffer, resuspended in 30 μL of 1×SDS-PAGE sample buffer, boiled for 10 min, and centrifuged for 2 min at 12,000 *g*. Ubiquitinated KLF5 was detected by Western blotting using the anti-Ub antibody.

### Immunohistochemical staining

To confirm the correlation between KLF5 and HDAC1 in breast tumor tissues, the samples were fixed with 4% buffered formaldehyde for 48 h at room temperature and embedded in paraffin. Paraffin-embedded clinical TNBC specimens were obtained from the First Affiliated Hospital, Zhengzhou University, Zhengzhou, China (Patient information is listed in Supplementary Table S1). Informed consent was obtained from all subjects. Two tissue microarrays containing 87 TNBC breast cancer tissues were constructed. For the immunohistochemistry (IHC) assay, the slides were deparaffinized, rehydrated, and pressure cooker heated for 2.5 min in EDTA for antigen retrieval. Endogenous peroxidase activity was inactivated by adding an endogenous peroxidase blocker (OriGene, Beijing, China) for 15 min at room temperature. Slides were incubated overnight at 4 °C with anti-KLF5 (1:1000) or anti-HDAC1 (1:2000). After 12 h, the slides were washed three times with PBS and incubated with secondary antibodies (hypersensitive enzyme-labeled goat anti-mouse/rabbit IgG polymer (OriGene, Beijing, China) at room temperature for 20 min, DAB concentrate chromogenic solution (1:200 dilution of concentrated DAB chromogenic solution), counterstained with 0.5% hematoxylin, dehydrated with graded concentrations of ethanol for 3 min each (70% - 80% - 90% - 100%), and finally stained with dimethyl benzene immunostained slides were evaluated by light microscopy. The IHC signal was scored using the 'Allred Score' method.

### Statistical analysis

Data are shown as mean ± standard deviation. The Student's t-test was used for statistical analysis. GraphPad Prism was used for all the statistical analyses. SPSS 20.0 software was used for Chi-square statistical analysis, The chi-square test was used to analyze the differences in HDAC1 and KLF5 expression among patients with different pathological grades. Fisher's exact probability test was used where the theoretical frequency was < 1. McNemar's test was used to compare the expression consistency of HDAC1 and KLF5 in patients. A two-sided test with a significance level of α = 0.05 was used for the statistical results. For the TNM stage comparison, we fit an ordinal variable of stage (I, II, and III) and estimated the relative risks (odds ratios) of one category increase in stage by triple-negative status. Repeated measures analysis of variance (RM-ANOVA) was applied to compare the cell viability of the time (0, 12, 24, 36 and 48 hours after intervention) and the effect of time-group interaction. The normality of distributions was verified using the Shapiro-Wilk test. All reported P-values were two-sided. Statistical significance was set at *P < 0.05; **P < 0.01; ***P < 0.001.

## Results

### TSA and SAHA suppressed BLBC cell growth, and promoted KLF5 acetylation, but downregulated the KLF5 protein levels

**Figure 1:**
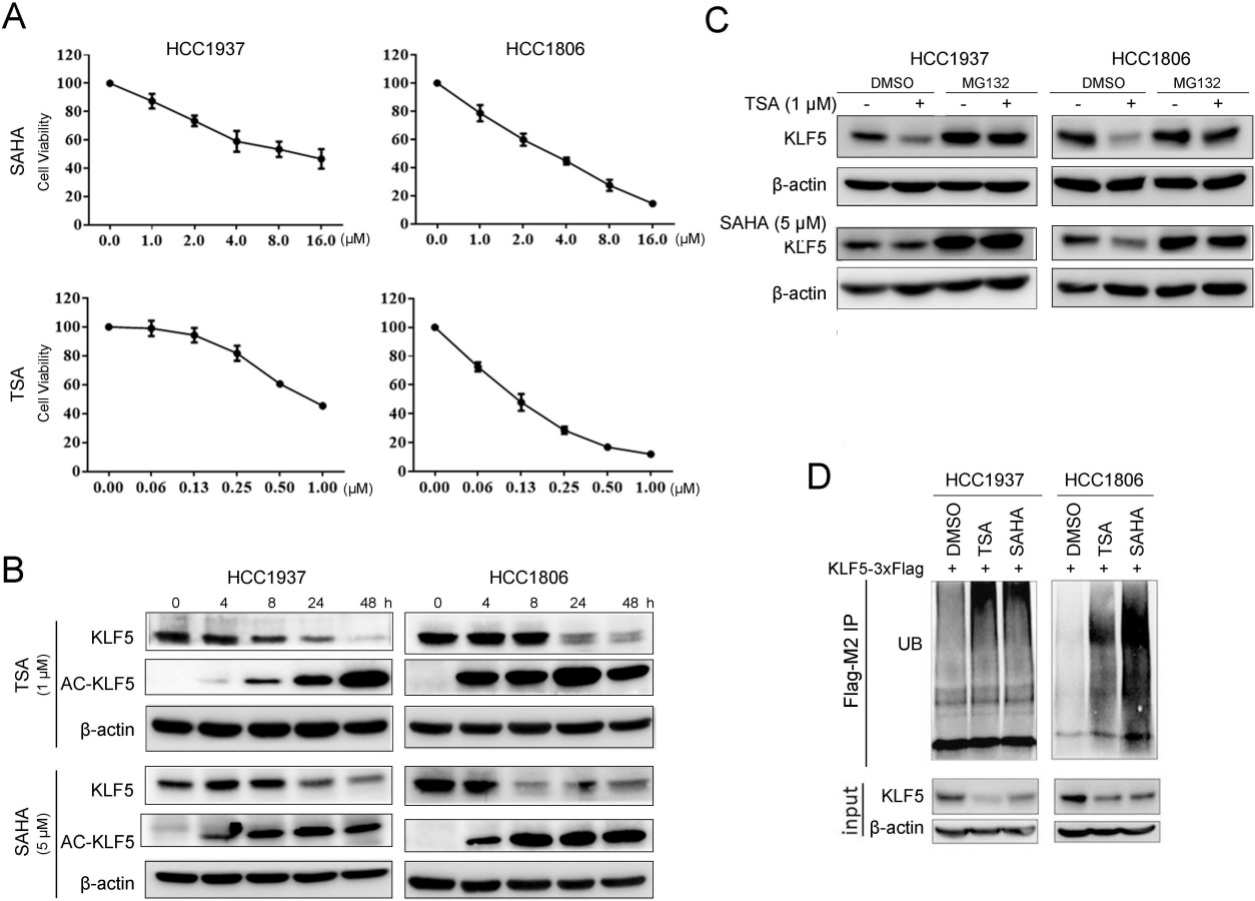
** HDACi inhibited KLF5 expression and cell viability in BLBC cell lines. A,** TSA and SAHA inhibited the HCC1806 and HCC1937 cell viability in a dose-dependent manner. TSA and SAHA were added to media at the indicated concentration, and cell viability was assessed after 48 h by sulforhodamine B (SRB) assays. **B,** TSA and SAHA decreased KLF5 protein expression in a time-dependent manner. HCC1937 and HCC1806 cells were treated with TSA (1 µM) and SAHA (5 µM) for different time periods (0, 4, 8, 24, 48 h), and KLF5 protein expression was analyzed by western blotting.** C,** the proteasome inhibitor MG132 blocked HDACi-induced KLF5 decrease in HCC1806 and HCC1937 cell lines. The cells were treated with TSA (1 µM) and SAHA (5 µM) for 8 h and then MG132 (3 µM) were added for 8 h. Cell lysates were collected for western blotting.** D,** these cells were treated with TSA (1 µM) and SAHA (5 µM) for 8 h and then MG132 (3 µM) were added for the next 8 h. Cell lysates were collected for western blotting.

HDACi are known to be cytotoxic to TNBC 49. We treated BLBC cell lines HCC1937 and HCC1806 with TSA and SAHA and found that the cell viability of both cell lines decreased in a dose-dependent manner. The IC_50_ values of SAHA in HCC1937 and HCC1806 were 8.3 μM and 5.1 μM, respectively; the IC_50_ values of TSA in HCC1937 and HCC1806 were 0.8 μM and 0.1 μM, respectively (Fig. 1A). Previous studies have shown that KLF5 promotes BLBC cell proliferation, survival, and migration 27 and that KLF5 is regulated by acetylation 50. We wondered whether HDACi regulated KLF5 expression in BLBC. In contrast to the reported results in the DU145 prostate cancer cell line 34, both TSA and SAHA reduced KLF5 protein expression levels in HCC1937 and HCC1806 cells in a time-dependent manner (Fig. 1B). To investigate whether HDACi promoted the acetylation of KLF5, we performed Western blotting with an anti-KLF5 K369 acetylation-specific antibody and found that HDACi treatment increased KLF5 acetylation at K369 (Fig. 1B). Next, we investigated the mechanism by which HDACi downregulate KLF5 protein expression in BLBC. We demonstrated that *KLF5* mRNA levels were not affected by SAHA or TSA treatment (Fig. S1A-B). Thus, it is possible that HDACi promote KLF5 protein degradation.

In previous reports, KLF5 was degraded by proteasomes 39, 51. When we treated BLBC cells with the proteasome inhibitor MG132, the HDACi-induced decrease in KLF5 levels was blocked (Fig. 1C). Furthermore, we showed that TSA and SAHA increased KLF5 ubiquitination (Fig. 1D).

### TSA and SAHA downregulated KLF5 and downstream target gene expression by inhibiting HDAC1

**Figure 2:**
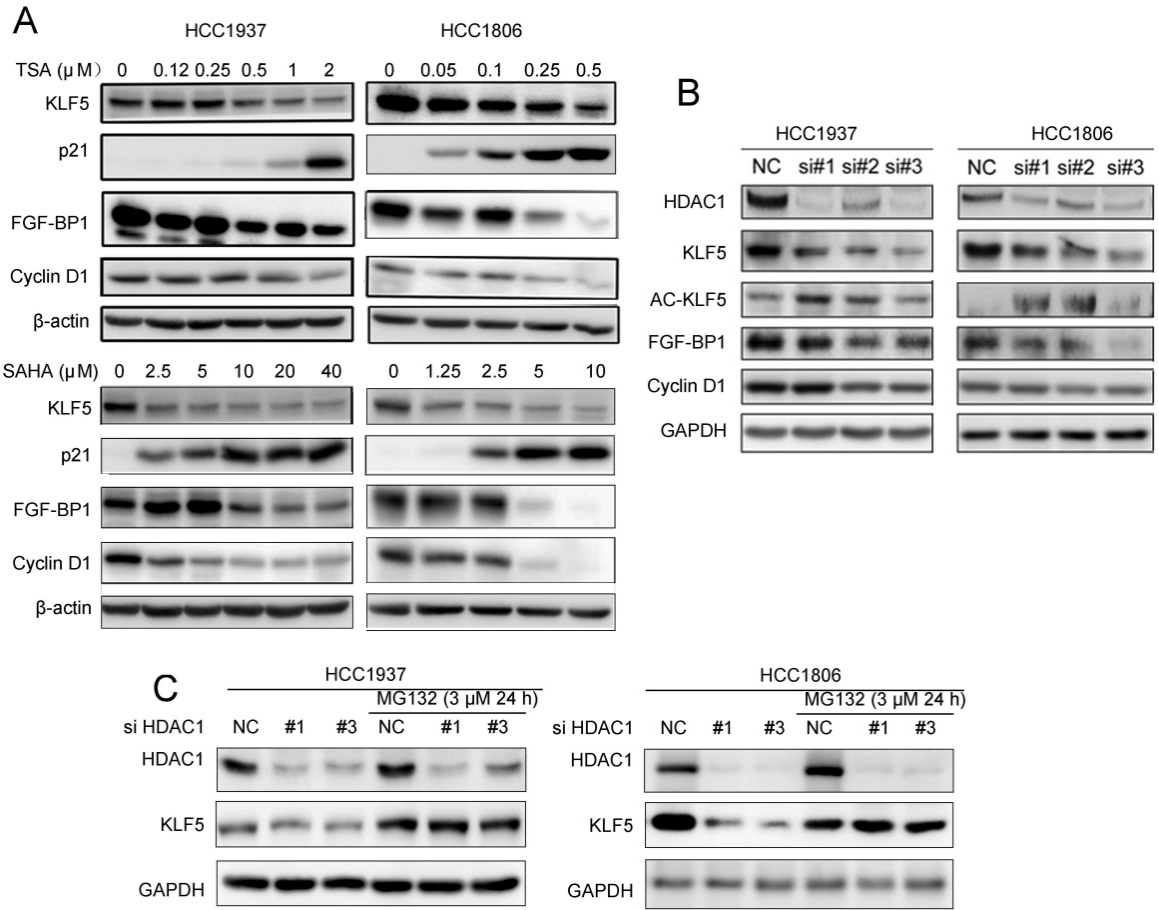
** Both HDACi and HDAC1 knockdown downregulated the expression of KLF5 protein and its downstream targets genes. A,** HDACi inhibited KLF5-mediated downstream target gene (FGF-BP1, Cyclin D1, and p21 expression changes. HCC1937 and HCC1806 cells were treated with HDACi for 48 h at the indicated concentrations. The protein expression was measured by Western blotting.** B,** HDAC1 knockdown by three different siRNAs in HCC1937 and HCC1806 cells for 48 h; cell lysates were collected for western blotting, which indicated that KLF5 and its downstream target gene (FGF-BP1 and Cyclin D1) expression were blocked.** C,** HDAC1 knockdown-induced reduction in KLF5 expression in BLBC was inhibited by proteasome inhibitor. HDAC1 was knocked down by three different siRNAs in HCC1937 and HCC1806 cells for 24 h. Subsequently, MG132 (3 µM) was used to treat the cells for 24 h. Cell lysates were collected for Western blotting.

Since HDACi down-regulate KLF5 expression in BLBC, we wondered whether HDACi suppress KLF5 transcriptional activity towards its downstream target genes. We treated HCC1937 and HCC1806 cells with TSA and SAHA, and measured the protein levels of KLF5 downstream target genes, including p21, FGF-BP1, and Cyclin D1. As expected, HDACi decreased the expression levels of FGF-BP1 and Cyclin D1, and increased the expression levels of p21 (Fig. 2A).

Both TSA and SAHA are inhibitors of class I deacetylases, and HDAC1 was reportedly responsible for KLF5 deacetylation in a previous study 50. We silenced HDAC1 with three different siRNAs and measured the protein levels of KLF5 and its downstream target genes. As shown in Fig. 2B, HDAC1 knockdown increased KLF5 acetylation and decreased the protein levels of KLF5 and its target genes, including *FGF-BP1* and *CCND1*, in both BLBC cell lines. When we added MG132 to HDAC1 knockdown groups, the decrease in KLF5 protein levels was inhibited (Fig. 2C). Furthermore, *KLF5* mRNA levels were not decreased by HDAC1 knockdown (Fig. S1C).

### KLF5 acetylation at K369 destabilized KLF5 protein

**Figure 3:**
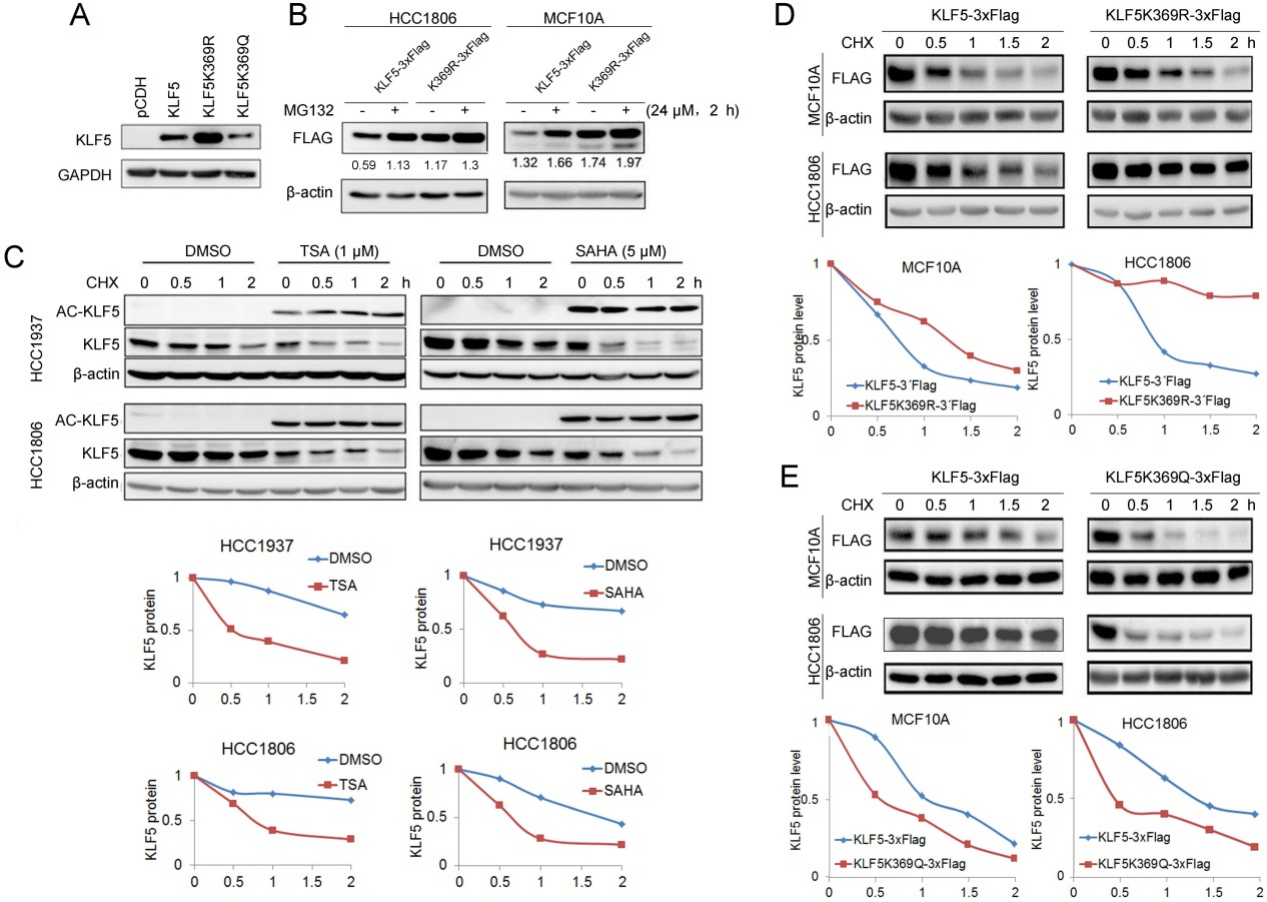
** KLF5 acetylation at K369 promoted its degradation. A,** WT KLF5, KLF5-K369R, and KLF5-K369Q were transfected in HEK293T cells. KLF5 expression levels were detected by Western blotting. **B,** KLF5-K369R was more stable than WT KLF5 protein in basal-like breast epithelial cells. WT KLF5-3×Flag and KLF5-K369R-3×Flag were stably overexpressed in HCC1806 and MCF10A cells. MG132 (24 μM) was added to treat the cells for 2 h. The KLF5/β-actin band intensity ratios are labeled below the blots.** C,** TSA and SAHA promoted the degradation of the KLF5 protein in HCC1937 and HCC1806 cells. The cells were treated with TSA (1 µM) or SAHA (5 µM) for 24 h. Then the cells were treated with cycloheximide (CHX, 50 μg/mL) for 0.5, 1, and 2 h. The cell lysates were collected for Western blotting. The quantitative results are plotted below. β-actin was used as the loading control.** D,** KLF5-K369R was more stable than WT KLF5 protein in basal-like breast epithelial cells. WT KLF5-3×Flag and KLF5-K369R-3×Flag were stably overexpressed in HCC1806 and MCF10A cells. The cells were treated with CHX (50 μg/mL) for 0.5, 1, and 2 h. The quantitative results are plotted below.** E,** KLF5-K369Q was less stable than WT KLF5 protein in basal-like breast epithelial cells. WT KLF5-3×Flag and KLF5-K369Q-3×Flag were stably overexpressed in HCC1806 and MCF10A cells. The cells were treated with CHX (50 μg/mL) for 0.5, 1, and 2 h. The quantitative results are plotted below.

It appears that KLF5 acetylation promotes ubiquitination and degradation of BLBC. The acetylation of KLF5 at K369 has been confirmed 50. To test whether KLF5 acetylation at K369 promotes KLF5 protein degradation, we overexpressed KR and KQ in HEK293T cells and found that the KR mutant showed a higher expression level than wild-type (WT) KLF5, while KQ mutant showed a lower expression level than WT KLF5 (Fig. 3A). Similarly, MG132 dramatically increased the WT KLF5 protein levels, compared with WT KLF5, MG132 slightly increased K369R protein levels in MCF10A and HCC1806 cells (Fig. 3B).

Next, we measured KLF5 protein stability using the CHX chase experiment to verify whether HDACi promote KLF5 protein degradation. As shown in Fig. 3C, both HDACi induced KLF5 acetylation and decreased the half-life of KLF5 (Fig. 3C). Subsequently, we investigated the half-lives of the KR and KQ mutant proteins. As shown in Fig. 3D-E, the KR protein half-life was prolonged compared to that of WT KLF5. In contrast, the half-lives of KQ protein were shorter than those of WT KLF5. These results indicate that KLF5 acetylation at K369 accelerated KLF5 protein degradation.

### KLF5 acetylation at K369 decreased the protein interaction between KLF5 and its DUB, BAP1

Our results suggest that K369 acetylation destabilizes KLF5 protein. It has been reported that KLF5 protein is ubiquitinated and degraded by WWP1 (enzyme activity mutant is WWP1C890A) 37, SCF^Fbw7^ (enzyme activity mutant is SCF^Fbw7ΔF^) 52 and Smurf2 38, but stabilized by BAP1 (enzyme activity mutant is BAP1C91S) 21, USP3 (enzyme activity mutant is USP3C186S) 43, ATXN3L 42, YAP (interaction with KLF5 mutant is YAP W1W2) 41, and TAZ (interaction with KLF5 mutant is TAZΔWW) 40. To characterize the mechanism by which K369 acetylation destabilizes KLF5, we first tested whether KLF5 E3 ligases could promote K369R mutant protein degradation. Three KLF5 E3 ubiquitin ligases (WWP1, Fbw7, and Smurf2) efficiently decreased KLF5 KR protein levels (Fig. S2).

**Figure 4:**
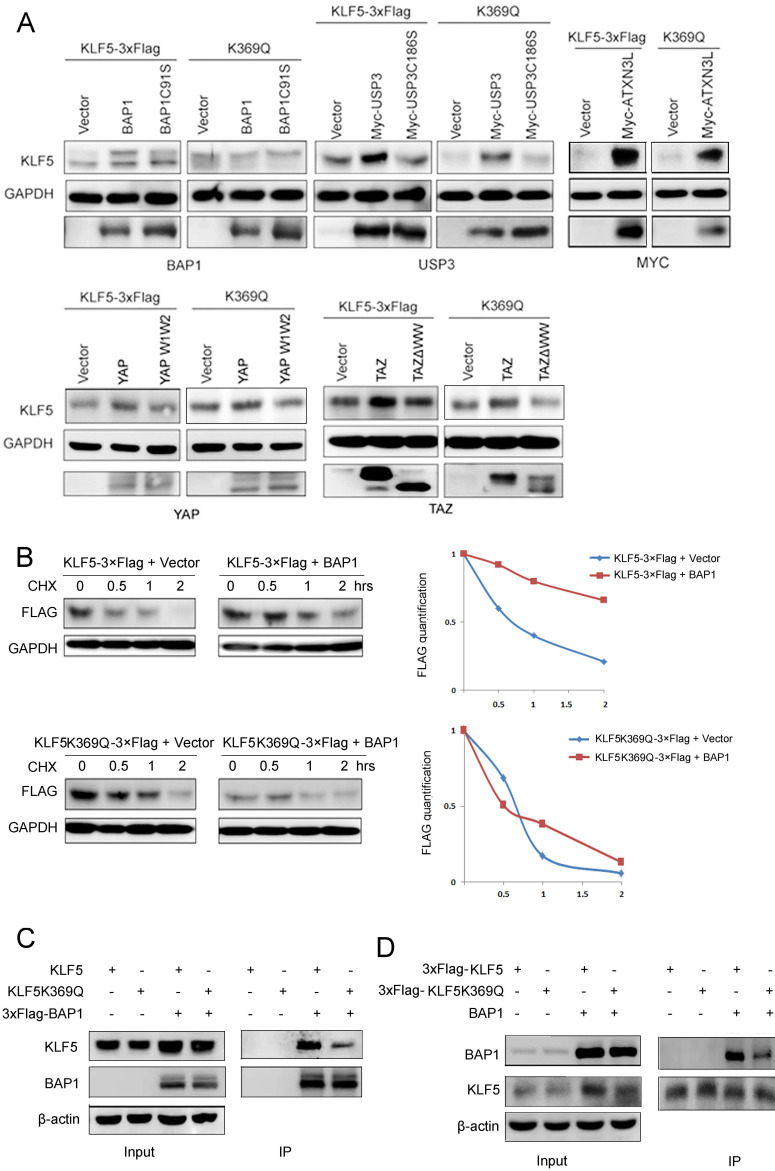
** KLF5 acetylation at K369 suppressed its interaction with BAP1. A,** BAP1 did not stabilize KLF5-K369Q protein. KLF5 and KLF5-K369Q were co-transfected with known KLF5 protein stabilizers (BAP1, USP3, ATXN3L, YAP, and TAZ) in HEK293T cells. Protein expression levels were detected by western blotting.** B,** BAP1 extended the protein half-life of WT KLF5, but not KLF5-K369Q. KLF5-3×Flag and KLF5-K369Q-3×Flag were co-transfected with empty vector and BAP1 in HEK293T cells. The cells were treated with CHX (50 μg/mL) for 0.5, 1, and 2 h. The quantitative results were plotted on the right.** C,** K369Q mutation decreased the protein interaction between KLF5 and BAP1. KLF5, KLF5-K369Q, and 3×Flag-BAP1 were co-expressed in HEK293T cells. 3×Flag-BAP1 was immunoprecipitated with anti-Flag-M2 beads. Protein expression levels were detected by Western blotting.** D,** K369Q mutation decreased the protein interaction between KLF5 and BAP1. KLF5-3×Flag, KLF5-K369Q-3×Flag, and BAP1 were co-expressed in HEK293T cells. KLF5-3×Flag and KLF5-K369Q-3×Flag proteins were immunoprecipitated with anti-Flag-M2 beads. Protein expression levels were detected by Western blotting.

Next, we tested whether KLF5 DUBs or YAP/TAZ could protect the KQ protein from degradation. We co-transfected BAP1, USP3, ATXN3L, YAP, and TAZ with WT or KQ mutant KLF5 into HEK293T cells and detected KLF5 protein levels. We demonstrated that only BAP1 could not efficiently protect KLF5^K369Q^ from degradation (Fig. 4A).

To investigate whether acetylated KLF5 protein escaped from BAP1-mediated stabilization, we performed CHX assays to measure the KLF5 protein half-lives. In agreement with our previous report 21, BAP1 efficiently extended the half-life of the WT KLF5 protein; however, BAP1 failed to extend the half-life of KLF5^K369Q^ (Fig. 4B).

We suspected that the acetylated KLF5 protein may decrease the interaction with BAP1. Indeed, BAP1 efficiently immunoprecipitated WT KLF5 protein, but not KLF5^K369Q^ (Fig. 4C). Consistently, KLF5^K369Q^ did not efficiently immunoprecipitate BAP1 (Fig. 4D). These results suggest that BAP1 does not efficiently interact with K369 acetylated KLF5; therefore, it does not protect it from degradation.

### KLF5 expression levels positively correlated with HDAC1 in TNBC tissues

Given that KLF5 directly interacts with HDAC1 53, 54, HDAC1 knockdown decreased KLF5 protein levels in BLBC cell lines (Fig. 2C). We wondered whether the expression levels of KLF5 and HDAC1 in TNBC tissues were positively correlated.

**Figure 5:**
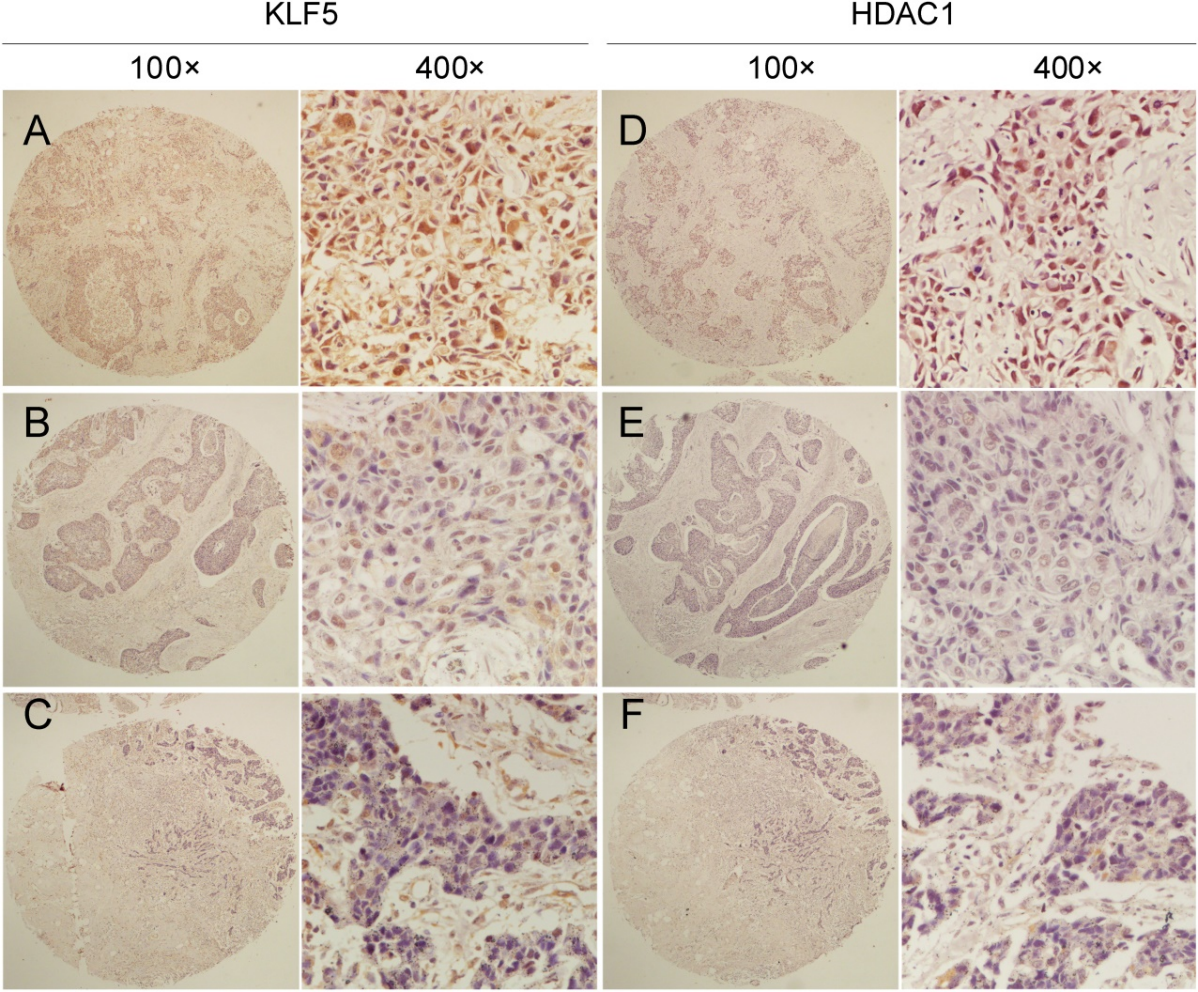
** The protein expression of HDAC1 and KLF5 were positively correlated in TNBC specimens.** Representative IHC images of KLF5 and HDAC1 protein expression in breast cancer tissues are shown. The final expression assessment was performed by combining the two scores (0-2=low, 3-5 = intermediate, 6-7 = high), A and D indicate high scores, B and E indicate intermediate scores, and C and F indicate low scores.

Clinic pathological parameters, including patient age, tumor size, lymph node status at the time of diagnosis, and follow-up status, including adjuvant treatment and tumor recurrence, were retrospectively obtained from the Department of Pathology, Henan Provincial People's Hospital, Zhengzhou University, Henan, China. We performed IHC analyses on two breast cancer tissue chips containing a total of 87 patients with TNBC (Table S1), showing that tumors with high KLF5 protein expression levels were significantly correlated with HDAC1 expression. McNemar's consistency test showed that the consistency of HDAC1 and KLF5 expression in patients with invasive ductal carcinoma was statistically significant (kappa=0.287, P<0.001). Spearman rank correlation analysis showed that HDAC1 and KLF5 expression were positively correlated (r = 0.379, P < 0.001) (Fig. 5 and Table 1).

### HDACi and BRD4i additively inhibit the KLF5 expression and BLBC cell growth

**Figure 6:**
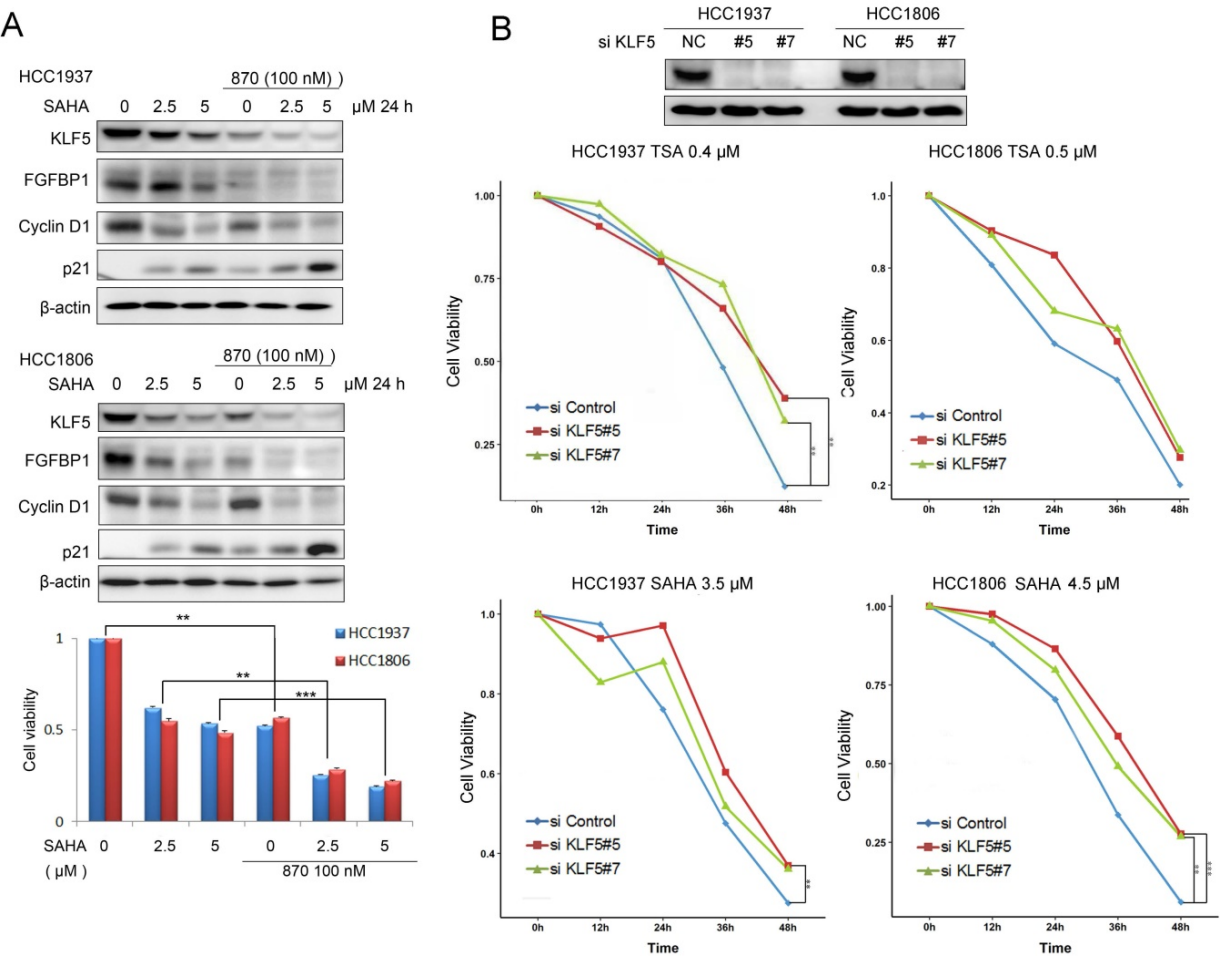
** HDACi in combination with BRD4i inhibit the KLF5 expression and function and the survival of BLBC cells. A,** HDACi were combined with compound 870 to regulate the expression of KLF5 and its downstream target genes (FGF-BP1, Cyclin D1, and p21). HCC1937 and HCC1806 cells were treated with HDACi and compound 870 for 24 h at the indicated concentrations. The protein expression was measured by Western blotting, and cell viability was assessed after 24 h by the SRB assay. **B,** KLF5 knockdown decreased the cytotoxicity of HDACi in BLBC cell lines. KLF5 was knocked down by two different siRNAs in HCC1937 and HCC1806 cells and cell lysates were collected for Western blotting. The cells were treated with HDACi for 48 h to measure cell viability by the SRB assay.

SAHA has been tested for TNBC patients in clinical trials in combination with chemotherapy. Since SAHA inhibits the KLF5 expression, we combined SAHA and BRD4 inhibitor 870 which was reported to inhibit KLF5 transcription in BLBC 27. We found that these two compounds additively decreased the expression of KLF5 and its target genes, including FGF-BP1 and CyclinD1, increased the expression of p21, decreased cell viability of BLBC cells (Fig. 6A).

To test whether HDACi function through KLF5, we knocked down KLF5 in HCC1937 and HCC1806 cells and treated the cells with HDACi. As shown in Fig. 6B, KLF5 depletion significantly decreased the efficacies of HDACi. These results suggest that HDACi inhibit BLBC through targeting KLF5, at least in part. As expected, HDACi inhibit BLBC also through other mechanisms in the absence of KLF5.

## Discussion

In this study, we demonstrated that HDAC inhibitors, TSA and SAHA, destabilized KLF5 protein and suppressed BLBC viability. We also showed that HDAC1 stabilized the KLF5 protein in BLBC cell lines. KLF5 acetylation at K369 promoted its ubiquitination and degradation because of its association with BAP1. These findings suggest that HDACi may be used to treat HDAC1-and KLF5-positive BLBC in the future.

Our conclusion is contrary to a previous report that knockdown of HDAC1/2 in the DU145 prostate cancer cell line promotes KLF5 acetylation at K369 but protects KLF5 from degradation 50. This could be attributed to the different cell types. We showed that KLF5 acetylation at K369 promoted ubiquitination. Most recently, it was reported that the growth of KLF5-K369R overexpression prostate cancer cells in nude mice is faster than WT KLF5 overexpression cells. Simultaneously, the growth of KLF5-K369Q overexpression cells is slower than WT KLF5 overexpression cells 55. These results support our hypothesis that KLF5-K369R is more stable and oncogenic *in vivo*. We found that KLF5^K369Q^ decreased its interaction with BAP1, a KLF5 DUB 21. We previously found that the KLF5 C-terminus (373-457) interacted with BAP1 21. Since K369 is very close to this region, it is reasonable that KLF5 acetylation at K369 could decrease its interaction with BAP1, although the exact mechanism requires further investigation. Previous studies reported that acetylation promoted protein degradation in hypoxia-inducible factor 1-alpha (HIF-1α) 46, GATA1 56, and retinoblastoma (Rb) proteins 47.

It is well known that TNBC are heterogeneous. TNBC was suggested to be classified into four different subtypes 57. KLF5 is highly expressed in basal-like breast cancers, including HCC1937 and HCC1806 cell lines, in contrast, KLF5 is lowly expressed in MDA-MB-231 cell line 20. HDACi also decreased the KLF5 protein expression levels in two different BLBC cell lines, such as BT549 and SUM149PT (Fig. S3), suggesting that inhibition of KLF5 by HDACi is a common phenomenon in BLBC.

It has been reported that KLF5 is positively regulated by the acetylase p300/CBP 54. GCN5 forms a complex with KLF5 and promotes KLF5 acetylation to facilitate lung cancer cell proliferation 58. Several deacetylases, including SET and HDAC1-4, have been reported to deacetylate KLF5 54, 59. In particular, HDAC1 can negatively regulate KLF5 through direct interaction, and inhibit the DNA-binding activity of KLF5 50, 54. Recently, HDAC3 was shown to interact with KLF5 to promote prostate cancer autophagy in response to docetaxel treatment 60. We tested whether the HDAC family (HDAC1-11, SIRT1-7) and HAT family (p300/CBP, GCN5, PCAF, and Tip60) could regulate KLF5 protein expression levels, and found that only HDAC1 stabilized KLF5 in BLBC (data not shown and Fig. 2).

Several HDACi are FDA-approved for the treatment of patients with hematological malignancies, e.g., romidepsin, vorinostat, and belinostat for T-cell lymphomas, and panobinostat for myeloma. In a phase III trial, postmenopausal women with HR^+^HER2^-^ advanced-stage breast cancer, resistant to endocrine therapy, benefitted from tucidinostat, a new HDACi 61. In clinical trials, SAHA was tested in combination with tamoxifen, olaparib, capecitabine (xeloda), paclitaxel, and bevacizumab against metastatic breast cancer 62. Carboplatin and nab-paclitaxel, with or without SAHA, have been tested for the treatment of women with newly diagnosed, operable breast cancer 63, 64.

HDACi suppress BLBC through KLF5, at least in part. After we depleted KLF5 in HCC1937 and HCC1806 cells, the inhibitory effects of HDACi were compromised (Fig. 6B). However, when we stably overexpressed KLF5 in HCC1937, HCC1806 and MCF10A, we could not rescue the loss of cell viability and apoptosis induced by HDACi (data not shown). We could not understand the mechanism at present. We observed that HDACi also decreased the c-Myc protein levels in these breast cell lines, suggesting that HDAC inhibitors may function through multiple targets besides KLF5.

In conclusion, TSA and SAHA promoted KLF5 protein degradation in BLBC by inhibiting HDAC1 activity and increasing KLF5 acetylation at K369, which abrogated its interaction with BAP1, a KLF5 DUB. Therefore, HDACi may hold potential for further development of anti-tumor regimens for BLBC.

## Supplementary Material

Supplementary figures and table.

## Figures and Tables

**Table 1:** Consistency and correlation of KLF5 expression among different HDAC1 expression groups

KLF5	HDAC1		Kappa	P (McNemar)	r	P (Spearman)
	Negative	Positive				
Negative	8 (9.1%)	23 (26.1%)	0.287	<0.001	0.379	<0.001
Positive	1 (1.1%)	56 (63.6%)				

## Abbreviations

BLBC: basal-like breast cancer
CHX: cycloheximide
DUB: deubiquitinase
GAPDH: glyceraldehyde 3-phosphate dehydrogenase
HDAC: histone deacetylase
HDACi: histone deacetylase inhibitors
IHC: immunohistochemistry
KLF5: Krüppel-like factor 5
KQ: acetylation-mimicking mutant KLF5^K369Q^
KR: acetylation-deficient mutant KLF5^K369R^
PARP: poly-ADP ribose polymerase
SAHA: suberoylanilide hydroxamic acid
SRB: sulforhodamine B
TNBC: triple-negative breast cancer
TSA: trichostatin A
WT: wild-type
