# Supplementary material for: Histone Deacetylase Inhibitors (HDACi) Promote KLF5 Ubiquitination and Degradation in Basal-like Breast Cancer
Source: Int J Biol Sci. 2022 Feb 28;18(5):2104–15. doi: 10.7150/ijbs.65322 (PMC8935240; doi:10.7150/ijbs.65322)
Supplement: Supplementary file 1 — Supplementary figures and table. [file ijbsv18p2104s1.pdf]

Supplementary Figures

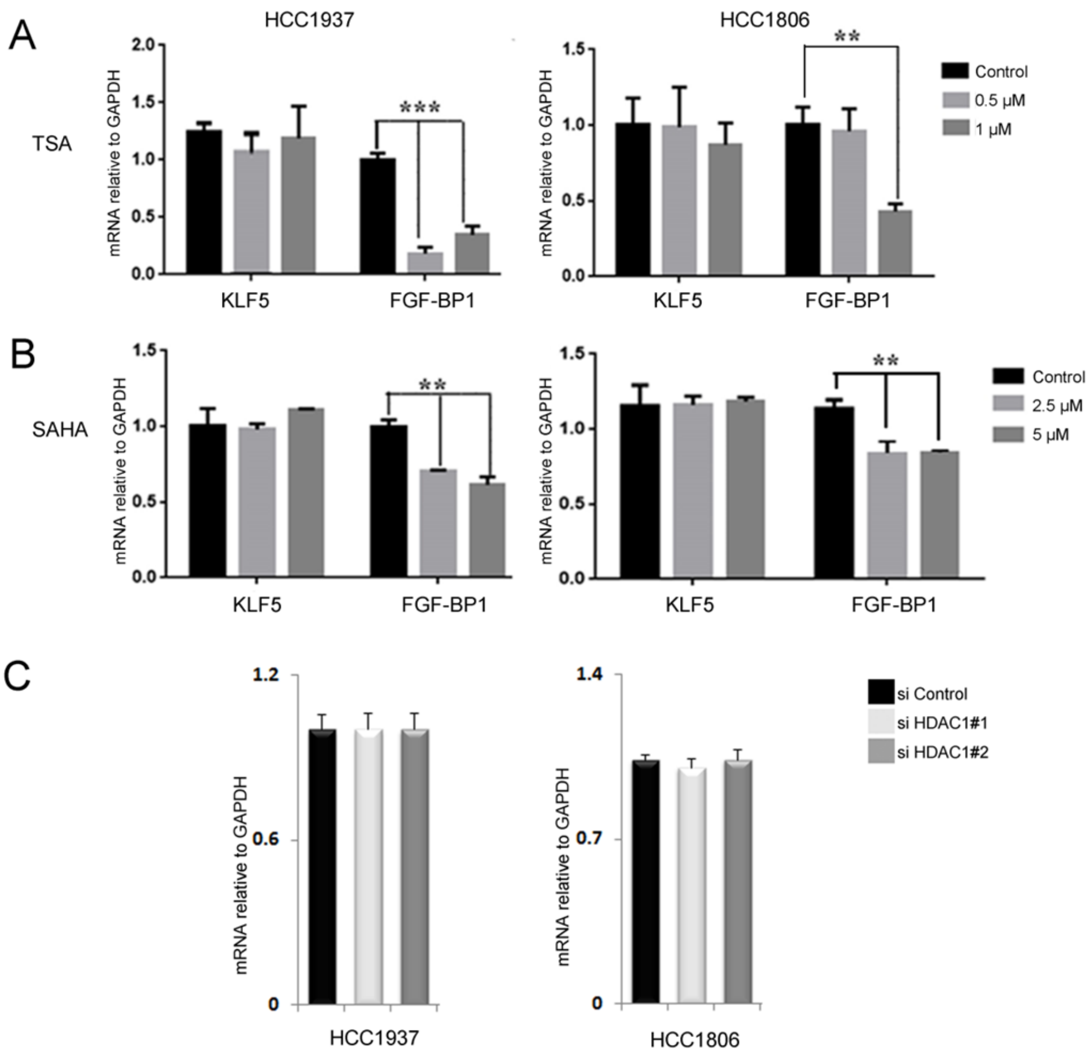

Supplementary Figure S1. TSA, SAHA, and HDAC1 knockdown did not decrease the *KLF5* mRNA levels in BLBC.

A. mRNA levels of *KLF5* and *FGF-BP1* in HCC1937 and HCC1806 cells after treatment with TSA, as detected by RT-qPCR. GAPDH was used as an internal control. $*P < 0.05$; $**P < 0.01$; $***P < 0.001$ (two-tailed Student's t test).

B. mRNA levels of *KLF5* and *FGF-BP1* in HCC1937 and HCC1806 cells after treatment with SAHA, as detected by RT-qPCR. GAPDH was used as an

internal control. *P < 0.05; **P < 0.01; ***P < 0.001 (two-tailed Student’s t test).

C. HDAC1 knockdown does not affect KLF5 mRNA levels in HCC1937 and HCC1806 cells. HCC1937 and HCC1806 cells were treated with HDAC1 siRNAs for 48 h. The mRNA levels of *KLF5* were detected by RT-qPCR. GAPDH was used as an internal control.

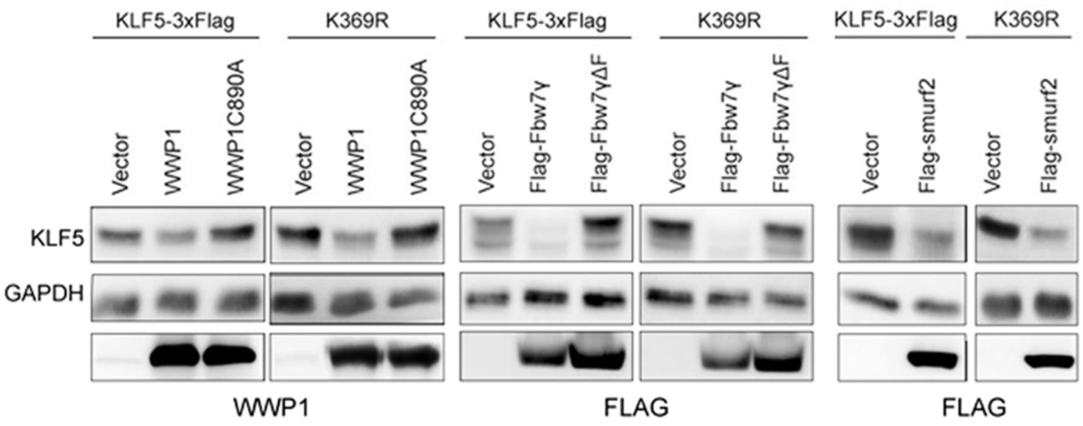

Supplementary Figure S2. KLF5 acetylation at K369 did not confer resistance to KLF5 E3 ligases, including WWP1, Fbw7γ, and Smurf2.

KLF5 and KLF5-K369R were co-transfected with KLF5 E3 ligases, including WWP1, Fbw7γ, and Smurf2, in HEK293T cells. Protein expression levels were determined by Western blotting.

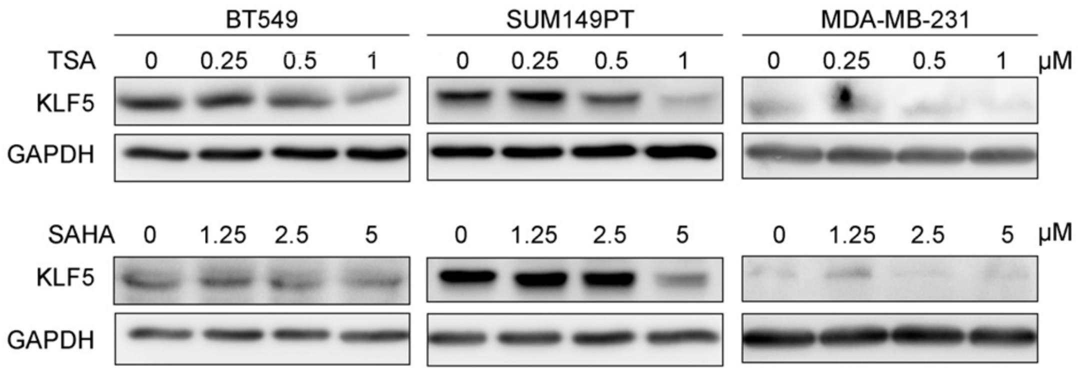

Supplementary Figure S3. HDACi inhibited KLF5 expression in TNBC cell lines. TSA and SAHA decreased KLF5 protein expression in a dose-dependent manner. BT549, SUM149PT and MDA-MB-231 cells were treated with TSA and SAHA for 24 h, KLF5 protein expression was analyzed by Western blotting.

Table S1 Patient and tumor characteristics (N=87)

Parameter	Values
Age (continous)	
Mean (SD)	48.2
Range	27-72
Tumor size	
T1 (0.1-2 cm)	18
T2 (2.1-5 cm)	61
T3 (5.1+ cm)	8
Tumor grade	
G1	3
G2	25
G3	59
TNM stage	
I	25
II	55
III-IV	7
Lymph node status	
Positive	30
Negative	57
